# Tobacco use and associated risk factors in Burkina Faso: results from a population-based cross-sectional survey

**DOI:** 10.1186/s12889-019-7826-6

**Published:** 2019-11-06

**Authors:** Bruno Bonnechère, Kadari Cissé, Tiéba Millogo, Gautier H. Ouédraogo, Franck Garanet, Mariam A. Ouedraogo, Gabriela Boyle, Sékou Samadoulougou, Seni Kouanda, Fati Kirakoya-Samadoulougou

**Affiliations:** 1 Centre de Recherche en Epidémiologie, Biostatistiques et Recherche Clinique, Ecole de Santé Publique, Université libre de Bruxelles, Lennik street 808, 1070 Brussels, Belgium; 2 Laboratory of Anatomy, Biomechanics and Organogenesis, Université Libre de Bruxelles, Brussels, Belgium; 3 Department of Electronics and Informatics, Vrije Universiteit Brussel, Brussels, Belgium; 4 imec, Leuven, Belgium; 5 Institut de recherche en sciences de la santé (IRSS), Ouagadougou, 03, BP 7102 Burkina Faso; 6 Institut Africain de Santé publique (IASP), Ouagadougou, 12, BP 199 Burkina Faso; 7 King County Public Health Department, Seattle, WA USA; 8 Evaluation Platform on Obesity Prevention, Quebec Heart and Lung Research Institute, Quebec City, Quebec Canada

**Keywords:** Tobacco consumption, Prevalence, Associated factors, Burkina Faso

## Abstract

**Background:**

Tobacco is a leading preventable cause of non-communicable diseases (NCDs). Studies characterizing the prevalence of tobacco use in low-income countries are lacking. This study describes the prevalence of tobacco use in Burkina Faso and its associated factors.

**Methods:**

Data from the 2013 Burkina Faso World Health Organization (WHO) Stepwise approach to Surveillance (STEPS) were analyzed. The prevalence of any tobacco product use, cigarette smoking, and other tobacco use was calculated. Logistic regression analyses identified factors associated with tobacco use. Overall, 4691 people were included in this analysis.

**Results:**

The prevalence of any tobacco use was 19.8% (95% CI: 18.4–21.2). Tobacco use was higher for men (29.2% [27.0–31.5]) than women (11.8% [10.3–13.4]). The prevalence of smoked tobacco was 11.3% (10.3–12.4), with a significantly higher prevalence among men (24.5% [22.1–27.0]) than women (0.1% [0.01–0.3]). The overall prevalence of other tobacco use was 8.9% (7.4–10.7), with lower values for men (5.6% [4.1–7.2]) compared to women (11.7% [9.4–14.1]). Tobacco smoking among men was significantly associated with an increased age and alcohol consumption. The analysis of risk factors for other tobacco use stratified by gender showed that age, education, residence, and alcohol consumption were significantly associated with consumption for women, and age and alcohol consumption for men.

**Conclusion:**

Tobacco use is common in Burkina Faso. To effectively reduce tobacco use in Burkina Faso, a comprehensive tobacco control program should consider associated factors, such as gender, age, and alcohol consumption.

## Introduction

Despite considerable efforts, tobacco use remains a leading cause of preventable deaths worldwide, according to the World Health Organization (WHO) [1]. Tobacco use, including smoking and the use of smokeless tobacco products (SLT), kills nearly 7 million people each year, including approximately 600,000 deaths from second-hand smoke [2–5]. If current trends persist, it is projected that tobacco use will kill over 8 million people per year by 2030, with 80% of these deaths occurring in low- or middle-income countries [6]. Characterizing tobacco use and associated factors is especially important for public health policy and intervention in low- and middle-income countries.

While the prevalence of tobacco use has been low in Africa compared to other regions, future projections show rapid increases in smoking, particularly among men, and an increase in the burden of non-communicable disease in the region, accentuating the need for country-level data on the prevalence of smoking and SLT use [7]. According to analyses of Demographic and Health Surveys (DHS), the prevalence of adult tobacco use smoking is highly heterogeneous across African countries, ranging from 7.6% in Ghana, 14.8% in Senegal, and 15.8% in Mali to 37.6% in Sierra Leone among men in Western Africa. The prevalence of smoking in all countries was higher in men than women, although studies have shown an increase in the marketing of tobacco products to women in sub-Saharan Africa (SSA) [8]. In most SSA countries, SLT consumption among women is higher than cigarette use [9].

In response to concerns about morbidity and mortality associated with tobacco consumption, the WHO created the Framework Convention on Tobacco Control (FCTC) in 2005 and “MPOWER” measures in 2008 as a set of low-cost, high-effective measures to control tobacco use [5, 10]. The use of population-based surveys, under the first pillar of the MPOWER strategy, helps countries and public health audiences to understand patterns of tobacco use and its associated factors, as well as to track the impact of tobacco control measures and policy changes [9].

According to 2011 Demographic and Health Survey data, the prevalence of smoking among a representative sample of adult men in Burkina Faso was 21.2% and among adult women, was 0.09%, with 3.86% reported SLT use among women. No SLT data was collected from men in this survey [10]. The only other nationally representative dataset that includes information about tobacco consumption in Burkina Faso is the 2013 WHO STEPwise approach to surveillance (STEPS) survey of non-communicable disease (NCD) risk factors. The present analysis uses these data to describe prevalence estimates for tobacco consumption in Burkina Faso and assess associated demographic and behavioral factors, in order to inform future prevention and control initiatives. In the context of the rapid increase in tobacco consumption predicted in SSA countries, it is particularly important to have accurate results on tobacco consumption in order to ensure an epidemiological surveillance program.

## Methods

### Data source

We analyzed cross-sectional data from the first Burkina Faso nationwide STEPS survey conducted in 2013 by the Ministry of Health of Burkina Faso with technical support from the WHO. STEPS is a population-based health survey administered to people aged 25–64 years in many countries worldwide that uses stratified three-stage cluster sampling proportional to size to select participants. The sample size is estimated using the following Schwartz formula: $n\ge deff\frac{Z_{\alpha}^2\ p\left(1-p\right)}{\varepsilon^2}\ast sub/\left(1-t\right)$ for p (high blood pressure prevalence previously estimated at 29.3%; deff design effect fixed at 1.5, *ε* absolute error (5%); *z*_*α*_ fractile of normal distribution of 5% error (1.96)) [11, 12]. The sample calculation was adjusted to account for a subgroup analysis of eight subgroups (*sub*) (four age groups, and two gender or two residence groups) and for a non-response rate (t) of 20%. The Burkina STEPS survey consisted of 4800 people, and 4691 participants were included in our analysis, after excluding observations with missing data about tobacco consumption. The response rate of the STEPS survey was 99.1% in Burkina Faso [12].

The study’s sampling frame was based upon enumerations (EAs) from the 2006 general census of the population and housing (GCPH) and updated in 2010 during the Demographic and Health Survey in Burkina Faso [13]. In the first stage, geographic areas were stratified into rural and urban, and EAs were selected with probability proportional to their size from both strata. A total of 240 EAs were selected: 185 from rural areas and 55 from urban areas. In the second stage, 20 households were selected from each EA. In the third stage, one person aged from 25 to 64 years in each household was selected using the Kish method.

The STEPS questionnaire is made up of several modules that include demographic information, anthropometric measures, and behavioral measurement. A full description of the study design and the data collection has been published elsewhere [13, 14]. All data about tobacco use and alcohol consumption were collected using a standardized questionnaire during face-to-face interviews.

The protocol of the STEPS survey was reviewed and approved by the Ethics Committee for Health Research of the Ministry of Health, which gave clearance in accordance with regulations in force (Deliberation No. 2012–12- 092 of 05 December 2012). Written informed consent was systematically sought and obtained from all participants before inclusion in the study. The confidentiality of study participants was fully respected and the analyses performed did not identify any participant.

### Study variables

Outcomes of interest include three measures of tobacco use: current any tobacco use, current cigarette smoking, and current other tobacco use (SLT). Current cigarette smoking was assessed with the question ‘During the past 30 days, how many days did you smoke cigarettes?’, with current smoking defined as smoking on at least 1 day during the past 30 days. Current SLT use was assessed with the question ‘During the past 30 days, on how many days did you use any smokeless tobacco products?’, with current SLT use defined as use of any SLT product on at least 1 day in the past 30 days. Any tobacco use is defined as either current cigarette smoking or other tobacco use in the past 30 days.

Independent variables included demographic characteristics such as age, sex, gender, marital status, place of residence, and education level, as well as behavioral measurements such as alcohol consumption (Table 1).

**Table 1:** Definition of recoded exposure variables

Variables	Categories
Age groups	“25 to 34 years old”, “35 to 44 years old”, “45 to 54 years old”, “55 to 64 years old”
Education	“None”, “Primary”, “Secondary/Tertiary”
Marital status	“Single”, “Married”, “Divorced/widowed”
Occupational status	“Wage earner”, “Self-employed”, “Jobless”
Alcohol consumption	None: Never intake of alcohol Low: intake of an average quantity of pure alcohol of less than 40 g per day for men and less than 20 g for women Mid: corresponds to taking an average quantity of pure alcohol of between 40 g and 59.9 g per day for men and between 20 g and 39.9 g for women High: intake of an average quantity of pure alcohol greater than or equal to 60 g per day for men and greater than or equal to 40 g for women.

### Statistical analysis

All data were weighted according to the cluster sampling design of the survey using strata and primary sampling units to allow the sample to be nationally representative. We used the Complex Samples module in Stata version 15 for data analyses. Weighted prevalence values (with corresponding 95% CIs) were calculated by sex and age group. We tested differences in prevalence with the χ^2^ test or Fisher exact test, when appropriate. Multivariable logistic regression models were used to estimate the associations between tobacco consumption, either smoking or other tobacco consumption, and other factors. A two-sided *p* value of less than 0.05 was considered statistically significant. We used QGIS to represent the variation of prevalence between the different regions of Burkina Faso.

## Results

### Socio-demographic characteristics

**Fig. 1:**
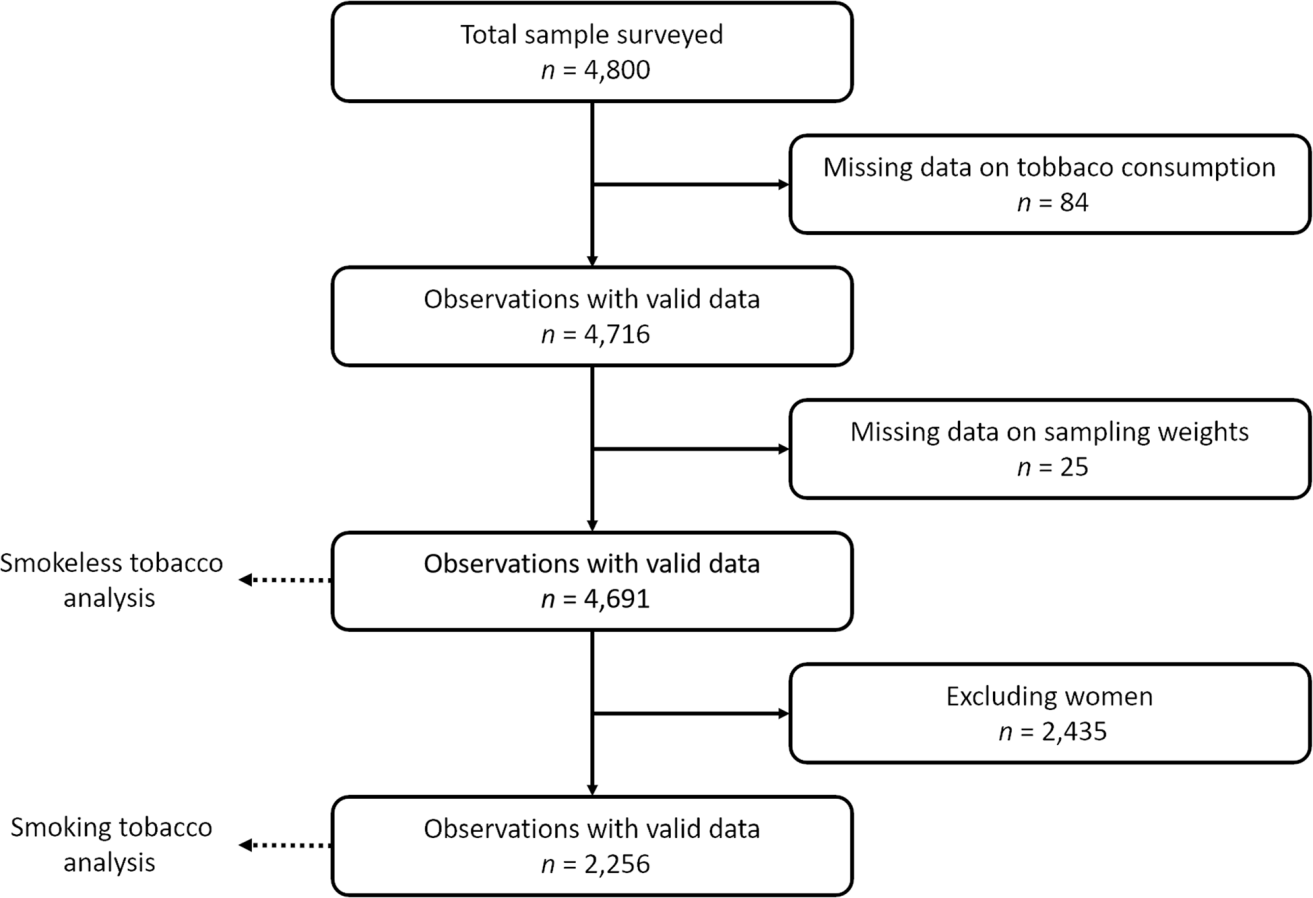
Diagram flow of study participants

Of the 4800 people originally sampled for the survey, 84 were excluded because they were not home after two visits, were not willing to participate in the study, or had no information available on tobacco use. The data of 25 people were deleted due to incomplete information about sampling weights in the database. The response rate for this analysis was 97.7%. Table 2 represents demographic characteristics of the sample and Fig. 1 shows the flow of the study participants. Of the 4691 people included in the analysis, men represented 48.1%. The age group of 25–34 years old had the highest percentage of participants (45.3%). Overall, 20.1% of participants lived in urban areas. Most participants had never gone to school (77.3%). Concerning the occupational status, 42% of women were housewives and 87.5% of men were self-employed.

**Table 2:** Socio-demographic characteristics of the study sample

Variables	Participants, n (%)	Women, n (%)	Men, n (%)
Age groups (*n =* 4691)			
25 to 34	2123 (45.3)	1175 (48.2)	948 (42.0)
35 to 44	1181 (25.2)	609 (25.0)	572 (25.3)
45 to 54	841 (17.9)	415 (17.1)	426 (18.9)
55 to 64	546 (11.6)	236 (9.7)	310 (13.7)
Education (*n =* 4683)			
None	3622 (77.3)	1974 (81.1)	1648 (73.3)
Primary	727 (15.5)	316 (13.0)	411 (18.3)
Secondary/Tertiary	334 (7.1)	145 (5.9)	189 (8.4)
Marital status (*n =* 4686)			
Single	333 (7.1)	75 (3.1)	258 (11.5)
Married	4042 (86.2)	2135 (87.8)	1907 (84.6)
Divorced/Widowed	311 (6.6)	223 (9.2)	88 (3.9)
Occupational status (*n =* 4691)			
Wage earner	281 (6.0)	86 (3.5)	195 (8.6)
Self-employed	3249 (69.2)	1274 (52.3)	1975 (87.5)
Jobless	1161 (24.7)	1075 (44.1)	86 (3.8)
Residence (*n =* 4691)			
Urban	1039 (22.2)	578 (23.6)	461 (20.4)
Rural	3652 (77.8)	1858 (76.4)	1794 (79.5)

### Tobacco use

**Fig. 2:**
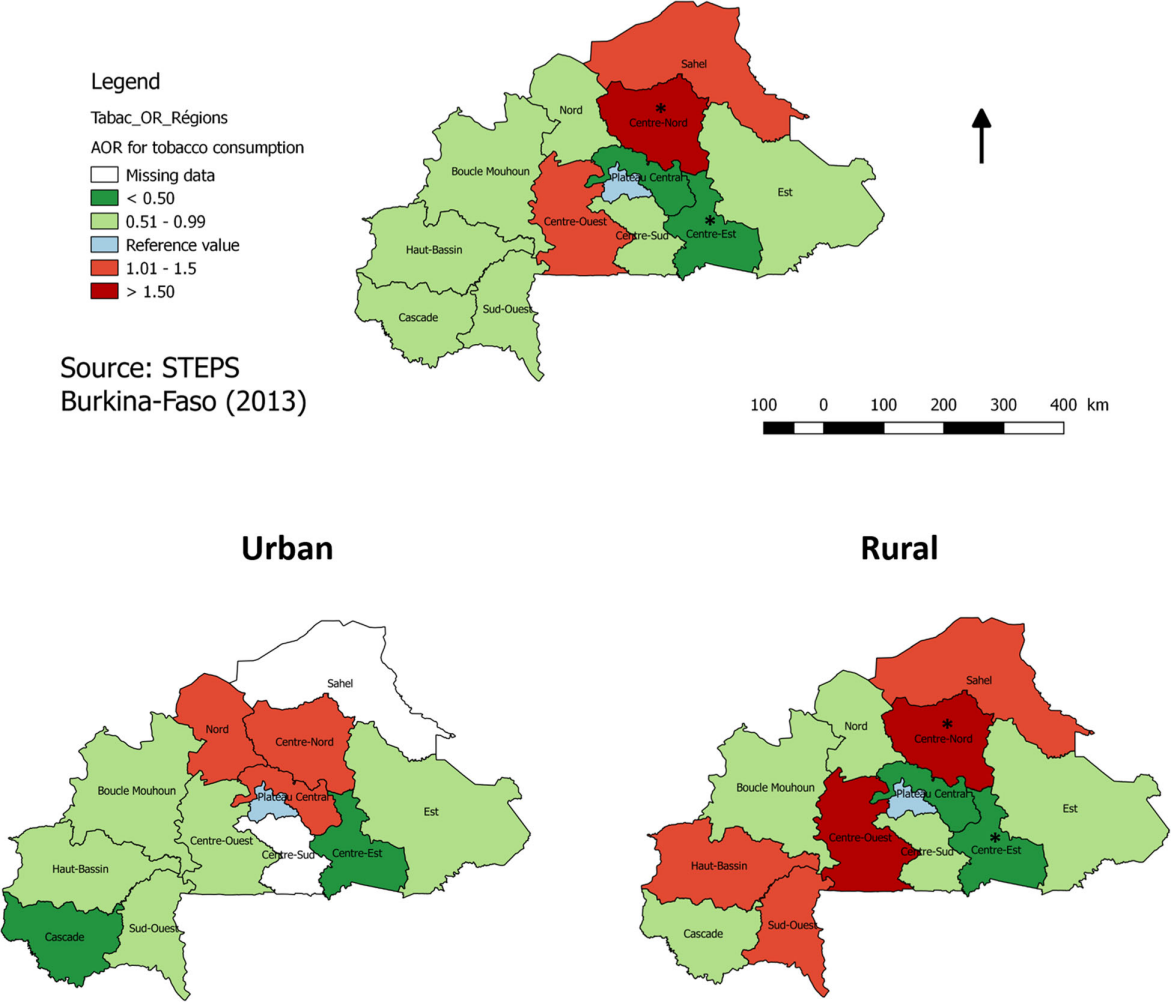
Adjusted OR (compared to the Ouagadougou region) for tobacco consumption in the different regions of Burkina Faso. Raw map has been downloaded and used with permission from GADM (https://gadm.org/) and updated with the result of this analysis

The overall prevalence of any tobacco consumption was 19.8% (95% CI: 17.8–21.9). The prevalence among males and females was statistically different, with a value of 29.2% (95% CI: 26.4–32.1) vs. 11.8% (95% CI: 9.6–14.3), respectively. Tobacco use was also statistically higher in rural areas (21.8% (95% CI: 19.4–24.3)) than in urban areas (14.1% (95% CI: 11.0–17.9, *p =* 0.001)). Tobacco use was highest in the Centre-Nord region (40.9% (95% CI: 29.3–53.6)) and lowest in the Centre-Est region (7.1% (95% CI: 3.7–13.2)). See Fig. 2 for the odd ratio (OR) of tobacco consumption in the different regions of the country.

Overall, 11.3% (95% CI: 10.3–12.4) of participants reported current smoking. The prevalence of smoking was also highest in the Centre-Nord region (17.3% (95% CI: 13.0–22.7)) and lowest in the Centre-Est region (3.9% (95% CI: 1.7–8.6)). The prevalence of daily smoking was 9.3% (95% CI: 8.3–10.6).

In total, 8.9% (95% CI: 7.4–10.7) of participants reported using smokeless tobacco (SLT) products. The Centre-Nord region had the highest prevalence of SLT use (25.2% (95% CI: 16.5–36.5)), and the Cascade region had the lowest prevalence (2.4% (95% CI: 0.7–7.8)). The prevalence of smoking was higher in men than in women (24.5% (95% CI: 22.1–27.0) vs. 0.07% (95% CI: 0.0–0.2), respectively). Women used SLT more frequently than men (11.7% (95% CI: 9.4–14.1) vs. 5.6% (95% CI: 4.1–7.2)). Smoking and SLT use were more frequent in rural areas than urban areas, with values of 11.6% vs. 10.4 and 10.8% vs. 3.7%, respectively. The prevalence of daily SLT use was 7.8% (95% CI: 6.5–9.5).

### Characteristics associated with tobacco use

The overall prevalence of tobacco smoking was 11.3% (95% CI: 10.3–12.4), but only two of the 2435 women included in this study reported smoking. Therefore, we limited the analysis of smoking and associated factors to the male population only (*n =* 2256). Table 3 presents the prevalence of smokers by characteristics and the results of univariate and multivariate analysis. The overall prevalence of cigarette smoking among men in Burkina Faso is 24.5% (95% CI: 22.1–27.0), with the highest prevalence in the 25–34 years age group (32.1% (95% CI: 28.3–36.2)). There is no statistically significant difference in smoking prevalence by living area (*p* = 0.79), education level (*p* = 0.22), marital status (*p* = 0.76), or occupational status (*p* = 0.07). Risk factors independently associated with tobacco smoking were sex (*p* < 0.001), age (*p* = 0.021), and alcohol consumption (*p* < 0.001). The prevalence of smoking decreases with age (Table 3) and there is a strong association between the levels of alcohol consumption and the risk of cigarette smoking.

**Table 3:** Risk factors for smoking tobacco consumptions in male population aged 25–64 years

Variable	n	Prevalence (95%CI)	Unadjusted OR (95%CI)	AOR (95%CI)
Age groups				
25 to 34	948	32.1 [28.3–36.2]	1	
35 to 44	572	23.0 [18.9–27.8]	0.62 [0.49–0.78]***	0.52 [0.38–0.72]**
44 to 54	426	19.1 [14.7–24.6]	0.45 [0.35–0.61]***	0.40 [0.27–0.60]***
55 to 64	310	13.6 [9.4–19.2]	0.37 [0.27–0.53]***	0.25 [0.16–0.39]***
Residence				
Urban	462	24.7 [20.0–30.1]	1	
Rural	1794	24.4 [21.7–27.3]	1.02 [0.81–1.30]	0.91 [0.63–1.30]
Education				
None	1648	23.9 [21.1–26.8]	1	
Primary	411	29.3 [23.2–36.2]	1.15 [0.90–1.47]	1.03 [0.75–1.41]
Secondary/Tertiary	189	19.4 [13.7–26.8]	0.70 [0.48–1.01]	0.69 [0.34–1.37]
Marital status				
Single	258	23.7 [17.7–30.9]	1	
Married	1907	24.4 [21.9–27.3]	1.01 [0.75–1.37]	1.36 [0.87–2.12]
Divorced/Widowed	88	26.6 [17.6–38.5]	1.27 [0.7–2.28]	1.72 [0.83–3.53]
Occupational status				
Wage earner	195	21.9 [15.9–29.1]	**1**	
Self-employed	1975	25.3 [22.7–28.1]	1.37 [0.96–1.97]	1.24 [0.70–2.19]
Jobless	86	11.6 [6.6–19.7]	0.62 [0.30–1.27]	0.45 [0.20–1.01]
Alcohol consumption				
No	1623	20.5 [18.0–23.2]	1	
Low	406	31.8 [25.5–38.9]	1.63 [1.28–2.07]***	2.01 [1.42–2.86]**
Mid	129	38.9 [29.1–49.6]	2.61 [1.80–3.77]***	2.87 [1.77–4.65]***
Abuse	98	40.3 [28.1–53.9]	2.39 [1.57–3.65]***	3.22 [1.74–5.94]***

*AOR* Adjusted Odds Ratios. *CI* Confidence interval, * *p* = 0.05, ** *p* = 0.01,*** *p* < 0.001

Bold highlights statistically significant variables

The overall prevalence of SLT consumption was 8.2% (95% CI: 7.4–8.9). The prevalence is 1.9% (95% CI: 1.2–3.1) in urban residents and 9.7% (95% CI: 8.8–10.7) in rural residents. The difference in SLT prevalence between living areas is statistically significant (*p* < 0.001) There is also a statistically significant difference between men and women (p < 0.001): the prevalence for women is 11.7% (95% CI: 9.4–14.1) and is 5.6% (95% CI: 4.1–7.2) for men. Among women, risk factors associated with SLT included age (p < 0.001), education level (*p* < 0.001), living area (*p* < 0.001), and alcohol consumption (*p* < 0.001) Among men, SLT use was associated with age (p < 0.001) and alcohol consumption (*p* < 0.001) (Table 4).

**Table 4:** Risk factors for smokeless tobacco consumptions in women and men population aged 25–64 years

Variables	Women			Men		
	n	Prevalence	AOR (95%CI)	n	Prevalence	AOR (95%CI)
Age groups						
25 to 34	1175	5.7 [3.2–10.1]	1	948	1.3 [0.6–2.5]	1
35 to 44	609	9.4 [6.8–12.9]	1.56 [1.01–2.41]**	572	2.2 [1.2–4.0]	1.52 [0.62–3.71]
44 to 54	415	23.5 [19.1–28.5]	4.47 [2.95–6.79] ***	426	11.3 [8.1–15.5]	8.32 [3.80–18.24]***
55 to 64	236	24.6 [18.7–31.6]	3.82 [2.27–6.41]***	310	17.0 [12.4–22.9]	12.88 [5.78–28.67]***
Residence						
Urban	578	3.8 [2.2–6.7]	1	461	3.5 [1.2–9.9]	1
Rural	1858	14.8 [12.0–18.1]	3.87 [2.09–7.13]***	1794	6.3 [4.8–8.2]	2.08 [0.83–5.21]
Education						
None	1974	13.8 [11.3–16.9]	1	1648	6.7 [5.1–8.7]	1
Primary	316	2.1 [0.8–5.5]	0.25 [0.09–0.70]**	411	2.5 [0.9–6.5]	0.45 [0.20–1.02]
Secondary/Tertiary	145	0.9 [0.2–1.6]	0.19 [0.03–1.39]	189	3.1 [0.7–13.1]	0.83 [0.17–4.12]
Marital status						
Single	75	0.8 [0.1–5.4]	1	258	1.4 [0.5–3.6]	1
Married	2135	11.0 [8.7–13.9]	3.40 [0.41–27.99]	1907	5.9 [4.5–7.7]	1.02 [0.34–3.12]
Divorced/Widowed	223	22.8 [16.4–30.9]	5.86 [0.68–50.54]	88	11.8 [3.9–30.4]	1.51 [0.32–7.21]
Occupational status						
Wage earner	86	0	/	195	0.9 [0.2–4.4]	1
Self-employed	1274	12.9 [9.4–17.6]	1	1975	6.1 [4.6–7.9]	**7.18 [1.01–51.31]**
Jobless	1075	11.2 [8.8–14.2]	1.00 [0.61–1.65]	86	6.0 [1.1–26.4]	16.00 [0.70–362.0]
Alcohol consumption						
None	1719	9.6 [7.5–12.2]	1.00	1623	4.0 [2.8–5.7]	1
Low	192	21.4 [15.4–29.0]	2.15 [1.34–3.46]***	406	8.5 [5.6–12.7]	2.17 [1.27–3.70]***
Mid	274	18.6 [13.1–25.7]	2.14 [1.37–3.35]***	129	9.4 [4.4618.9]	3.04 [1.27–7.25]**
Abuse	30	20.3 [7.1–45.7]	2.19 [0.58–8.28]	98	14.9 [7.7–26.8]	4.10 [1.72–9.74]***

*AOR* Adjusted Odds Ratios. *CI* Confidence interval, * *p* = 0.05, ** *p* = 0.01,*** *p* < 0.001

Bold highlights statistically significant variables

## Discussion

Our study analyzed the most recent national representative survey data on tobacco consumption among adults in Burkina Faso and our findings suggest that tobacco consumption is higher than what was reported from the 2011 DHS survey [9]. It is not possible to compare our findings with others, such as the age-standardized prevalence of daily smoking from 2015, which considers daily smoking instead of any current smoking and found the age-standardized prevalence of daily smoking for 2015 women to be 4.2% and for men, to be 12.5% [4].

From 2006 to 2013, the government of Burkina Faso carried out national tobacco control programs. Laws regulate tobacco consumption health care facilities, educational facilities, government facilities, and indoor offices. Additionally, Burkina Faso has national bans on the direct advertising of tobacco products on billboards and outdoor advertising, as well as on national television or radio [5]. In addition, an addiction center was recently opened in the largest hospital in the country. Despite national action to decrease tobacco use since the WHO FCTC took effect on October 2006, Burkina Faso continues to have higher tobacco consumption than other countries in the region [5, 9, 15]. The results from our analyses of STEPS data, as well as the 2011 DHS data, show that Burkina Faso’s smoking prevalence in men is higher than seven of nine other countries in West Africa and the prevalence of SLT in Burkina Faso is higher than nine of 10 other countries in West Africa [9]. The adoption and implementation of tobacco control measures has been slow and faced major challenges in Burkina Faso compared to some countries where vigorous measures have been adopted. The adoption of strict regulatory texts and measures against smoking has been recorded in recent years. The last of these texts is the decree No. 2015–366/MS/MICA fixing the modalities of packaging and labeling of tobacco products in Burkina Faso. This text recommends that importers and manufacturers of tobacco affix graphic health markings on tobacco packets. The government must strive for effective communication in the fight against tobacco within the population and the strict application of current regulations to address the prevalence of smoking. Young people are the main consumers of tobacco in Burkina Faso. Our findings show that nearly one of third of young people aged 25–34 years-old reported smoking tobacco and that prevalence decreases with age. In pooled data from 30 SSA countries, age is associated with tobacco consumption; however, both smoking and SLT use increase with age [9]. However, analyses of STEPS data from Kenya also found that the majority of smokers were in younger age groups [16]. Earlier smoking initiation is a major public health concern. The average age of smoking initiation among adults in Burkina Faso was 20.9 years of age, highlighting the importance of tobacco prevention policies to address people in younger age groups [12]. The Global Youth Tobacco Survey carried out in two cities in Burkina Faso in 2009 found that about 11.9% of boys from 13 to 15 years old in Ouagadougou and 6.0% in Bobo-Dioulasso were currently tobacco smokers [17, 18]. While youth smoking has decreased between 2001 and 2009 in both of these cities, the prevalence of the use of other tobacco products increased, as did youth reports of exposure to second-hand smoke at home in Bobo-Dioulasso [17].

In our study, smoking was also significantly associated with gender and alcohol consumption, but not to location of residence. In various other SSA countries, tobacco use is higher in men than in women [9], likely related to differing social norms about gender and tobacco use. Alcohol consumption and tobacco use were also linked in Kenya’s STEPS survey [16], among other studies. The combination of those two risk factors may contribute to future increases of NCDs in Burkina Faso. Our study did not find any association between smoking, education, or employment status, while other studies from SSA found that men and women from rural areas, or those with lower educational levels, smoke more than those from urban areas [9].

According to our findings, SLT is more frequently used by women and among those living in rural areas of Burkina Faso. Although SLT is less prevalent than smoking, it presents an important public health problem due to its association with many diseases, such as cancer (i.e., mouth, pharynx, and esophagus cancer) and ischemic heart disease. Health effects of SLT vary by region, related to the types of tobacco that are used in those regions [19]. In Sub-Saharan Africa, few studies have focused on the health effects of SLT [20, 21]. In general, women SLT users are exposed to multiple health risks, such as pregnancy complications (e.g., placenta praevia, placental abruption, and pre-eclampsia) [22]. In this study we did not estimate SLT consumption during pregnancy. A study published in 2017, however, found that the prevalence of SLT during pregnancy in Burkina Faso was 2.8%. This prevalence is higher compared to other African regions (1.7%), but lower than in Sierra Leone (4.6%) [22]. Even at a national level, as shown in Fig. 2, important differences were observed between the regions. Therefore, further and more advanced spatial analysis of tobacco consumption is needed to better guide health care prevention programs.

Concerning the limitations of this study, we report here the results of the first nationally representative survey on the prevalence and risk factors for tobacco consumption in Burkina Faso. The first limitation stems from the cross-sectional nature of the data, which limits the possibility of deriving causal inferences. The second limitation is that tobacco and alcohol consumption were obtained during interviews and are therefore dependent on the faith of the participants. There is thus both a risk of memory bias and social desirability. It can therefore be estimated that the numbers and prevalence obtained in this survey underestimate the actual consumption. The last point is that some well-known risk factors for tobacco consumption were not included in the study because data on these variables have not been collected during the STEPS survey. One of these variables is the socio-economic status. Given the study design (cluster sampling design) and the sample size, the results of this study can be extended to the whole of Burkina Faso.

Based on the results of this study, important preventive measures need to be taken to reduce tobacco consumption in Burkina Faso, with targeted approaches to sections of the population most affected by different types of tobacco use. In general, efforts should target tobacco use in youth, smoking among men, and SLT consumption among women and in rural areas.

Two important aspects should be taken into consideration when organizing tobacco prevention campaigns in Burkina Faso. The first is the necessity to integrate tobacco and alcohol campaign programs, since we have seen that both consumptions are highly correlated for both smoked and SLT consumption. The second is to pay more attention to SLT use in tobacco control programs as it constitutes a significant part of the tobacco use in Burkina Faso and is the principal type found in women.

## Conclusion

The prevalence of tobacco consumption remains high in Burkina Faso, despite restrictive measures and control plans adopted in recent years. Since tobacco consumption is an important risk factor for cardiovascular diseases and other NCDs, it is important to promote and accelerate the implementation of various measures to decrease consumption, especially SLT consumption, in future years. Further spatial analyses could be useful to identify areas of high tobacco consumption in order to focus more on these areas in tobacco control and prevention program planning.

## Data Availability

The dataset of the STEPS survey that was used in this research is available at the Ministry of Health upon request. Any request to reanalyze the data can be directed to Dr. Brice Bicaba.

## Abbreviations

DHS: Demographic and Health Surveys
NCD: Non-communicable disease
SLT: Smokeless tobacco products
SSA: Sub-Saharan Africa
STEPS: WHO STEPwise approach to surveillance
WHO: World Health Organization
